# Rising co-payments coincide with unwanted effects on continuity of healthcare for patients with schizophrenia in the Netherlands

**DOI:** 10.1371/journal.pone.0222046

**Published:** 2019-09-12

**Authors:** Arnold P. M. van der Lee, Lieuwe de Haan, Aartjan T. F. Beekman

**Affiliations:** 1 Department Psychiatry Amsterdam University Medical Centre–location VUmc, Amsterdam, The Netherlands; 2 Department Psychiatry Amsterdam University Medical Centre–location AMC, Amsterdam, The Netherlands; University of Sydney, AUSTRALIA

## Abstract

**Background:**

Co-payments, used to control rising costs of healthcare, may lead to disruption of appropriate outpatient care and to increases in acute crisis treatment or hospital admission in patients with schizophrenia. An abrupt rise in co-payments in 2012 in the Netherlands offered a natural experiment to study the effects of co-payments on continuity of healthcare in schizophrenia.

**Methods:**

Retrospective longitudinal registry-based cohort study. Outcome measures were (i) continuity of elective (planned) psychiatric care (outpatient care and/or antipsychotic medication); (ii) acute psychiatric care (crisis treatment and hospital admission); and (iii) somatic care per quarter of the years 2009–2014.

**Results:**

10 911 patients with schizophrenia were included. During the six-year follow-up period the level of elective psychiatric outpatient care (-20%); and acute psychiatric care (-37%) decreased. Treatment restricted to antipsychotic medication (without concurrent outpatient psychiatric care) increased (67%). The use of somatic care also increased (24%). Use of acute psychiatric care was highest in quarters when only antipsychotic medication was received. The majority (59%) of patients received continuous elective psychiatric care in 2009–2014. Patients receiving continuous care needed only half the acute psychiatric care needed by patients not in continuous care. On top of these trends time series analysis (ARIMA) showed that the abrupt rise in co-payments from 2012 onwards coincided with significant increases in stand-alone treatment with antipsychotic medication and acute psychiatric care.

**Conclusions:**

The use of psychiatric care decreased substantially among a cohort of patients with schizophrenia. The high rise in co-payments from 2012 onwards coincided with significant increases in stand-alone treatment with antipsychotic medication and acute psychiatric care.

## Introduction

Schizophrenia is a serious mental illness and a chronic disease for most patients. Patients with schizophrenia often suffer from somatic comorbidities and have a 15–25 year reduced life-expectancy[1–9]. Patients fare best when in continuous, integrated healthcare, which consists of psychiatric and somatic care. This may help to prevent psychotic relapse, crisis treatment, hospitalization, and early mortality[10–25].

The Netherlands has a well-established care system for patients with schizophrenia[26–31]. Patients can choose any healthcare provider. All inhabitants in The Netherlands have compulsory health insurance and are free to choose and have to be accepted by any health insurance company (regardless of their health or income)[[31]]. 73% of patients in a cohort received continuity of care in 2009–2011[32]. Rising costs of healthcare in The Netherlands, but also in many other countries, led to re-evaluation of the way healthcare is financed[30,33–35]. The Netherlands has a long-standing policy with universal access to a wide range of evidence-based mental health treatments and few financial barriers to such care. Co-payments for patients in The Netherlands were traditionally modest compared to other countries but are rising[36]. In the period between 2012 and 2013 health-related co-payments were raised significantly, for everyone, from €155 to €360 euro. In addition, during 2012 a separate co-payment for psychiatric care was charged[37,38]. Although there is evidence that the effects of co-payments are smaller among patients with severe health problems, there is also evidence that effects are larger among patients with mental disorders[39–42]. Continuity of elective outpatient care is essential for patients with schizophrenia. Co-payments may disrupt this. Unwanted effects may include a rise in use of acute psychiatric care (inpatient or crisis care).

The introduction and rise of co-payments offered a natural experiment to study the effects of co-payments on healthcare use of patients with schizophrenia. Other factors than co-payments which influence healthcare use have to be considered, for instance the national policy to substitute inpatient care with elective planned outpatient treatment[43]. A previous paper has shown that, overall, the use of mental healthcare declined substantially after the introduction of co-payments and that this effect was stronger for patients with lower incomes[44]. Patients with schizophrenia often have very low incomes[40–42]. Patients with schizophrenia may be even more vulnerable to unwanted effects of co-payments because a substantial proportion of schizophrenia patients has decreased insight in their illness and/or is reluctant to accept treatment.

We expected that (i) overall there would be a decline in the long-term use of psychiatric care and (ii) tested whether there were significant deviations on top of the long-term declining trend that correlated temporally with the abrupt rise in co-payments in 2012 in the Netherlands. The deviations we expected were a relative decrease in continuous outpatient psychiatric care and a relative increase in acute psychiatric care (inpatient or crisis treatment) among patients with schizophrenia.

## Methods

### Study design and patient selection

Computerized registry data of the largest Dutch health insurer (Zilveren Kruis) were collected for all insured persons. All patients insured by Zilveren Kruis with a diagnosis of schizophrenia in 2008 under 70 years of age were selected. Mental and somatic healthcare use, including prescription data of antipsychotic medication, of these patients over 2009–2014 were analyzed in a retrospective, longitudinal registry-based cohort study.

Zilveren Kruis provided health coverage for about 30% of the 16.4 million residents in the Netherlands in 2008. Those insured by Zilveren Kruis were representative of the Dutch population.

**Fig 1:**
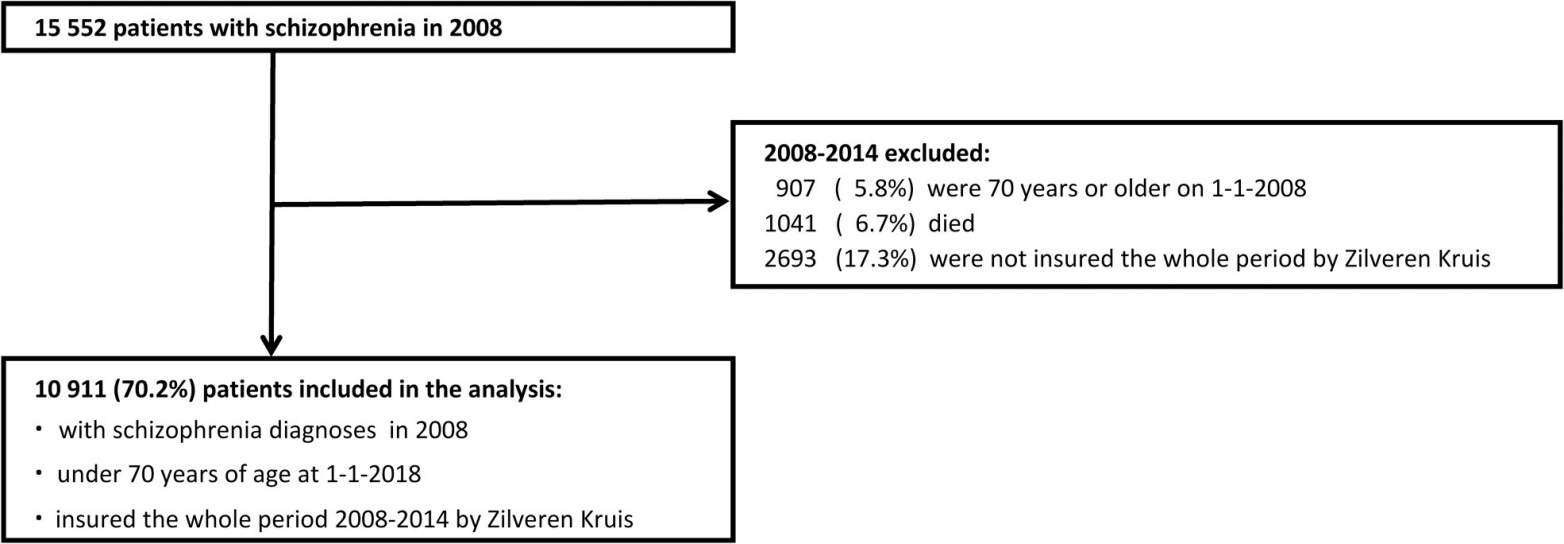
Selection of 10 911 patients with schizophrenia included in the study cohort.

In 2008, the first year mental health care was provided under the Dutch Health Insurance Law, there were 15 552 patients who had claims with the diagnosis schizophrenia (Fig 1).

Excluded were: 907 (5.8%) patients who were 70 years or older on January 1 2008, 1041 (6.7%) patients who died and 2 693 (17.3%) patients who were not insured the whole period by Zilveren Kruis. 10 911 (70.2%) patients, under 70 years of age and insured during the whole study period by Zilveren Kruis, were included in the analysis. 61% were men (mean age 40.2, SD 11.4) and 39% were women (mean age 46.0, SD 12.3).

### Data source: Dutch computerized health insurance registry data

All data are derived from Zilveren Kruis health insurance registry data. Dutch health insurance companies thoroughly process and pay the claims for all healthcare which is covered by the Dutch Health Insurance Law. The claims process is regulated by the National Care Authority. Dutch health insurers have implemented and maintain very strict rules and regulations about privacy of their insured and their healthcare providers according to prevailing law in the Netherlands. The selection and analysis of the necessary data for this study took place according to these rules. The analysis was carried out with data through which individual patients could not be identified. Therefore, no informed consent nor approval of a Medical Ethical Committee was needed. The Zilveren Kruis database contains data concerning all the care, as covered by the Dutch Health Insurance Law, received by these patients from all their healthcare providers.

The information concerning diagnoses is limited. The main groups of DSM-IV diagnoses are registered in the registry, not the detailed codes of the schizophrenia spectrum. The diagnoses of schizophrenia in this study were registered by the treating psychiatrist and pertain to the time of inclusion and the period covered by the study. Although registry based diagnoses can be unreliable, recent work shows that diagnoses of schizophrenia are sufficiently reliable for use in in study such as ours[45]. Separate treatments for more than one psychiatric disorder will result in separate claims. For short-term treatment no diagnosis has to be provided. A psychiatric crisis situation has to be registered on the claim. The maximum duration for such a claim is 28 days, for all other claims the maximum duration is one year. The claims for outpatient medication, which were prescribed and picked up at the pharmacy, include the name, price, and the Defined Daily Dose (DDD) of every medication. Information on inpatient medication is not available in the registry dataset.

### Co-payments

**Fig 2:**
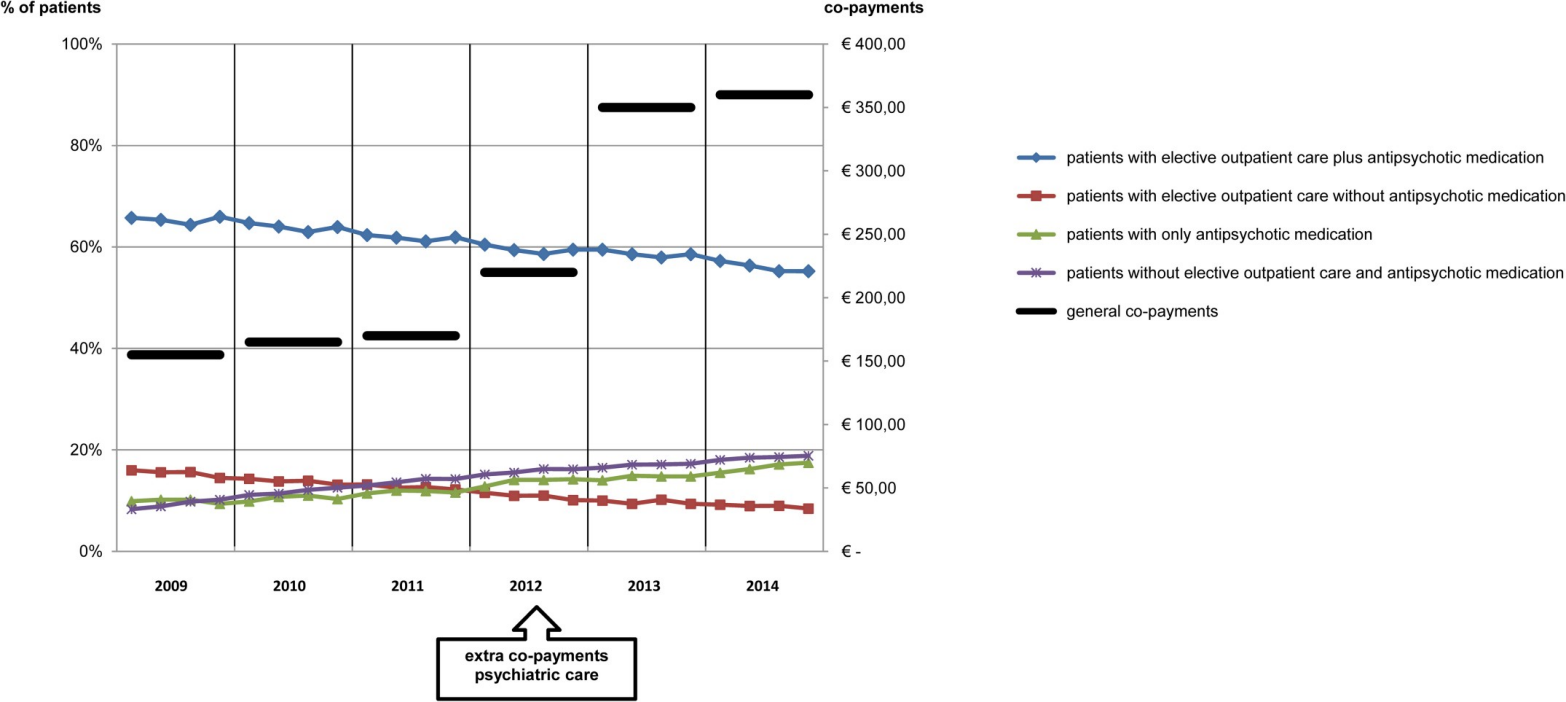
Elective psychiatric care for patients with schizophrenia in relation to co-payments for psychiatric care.

**Fig 3:**
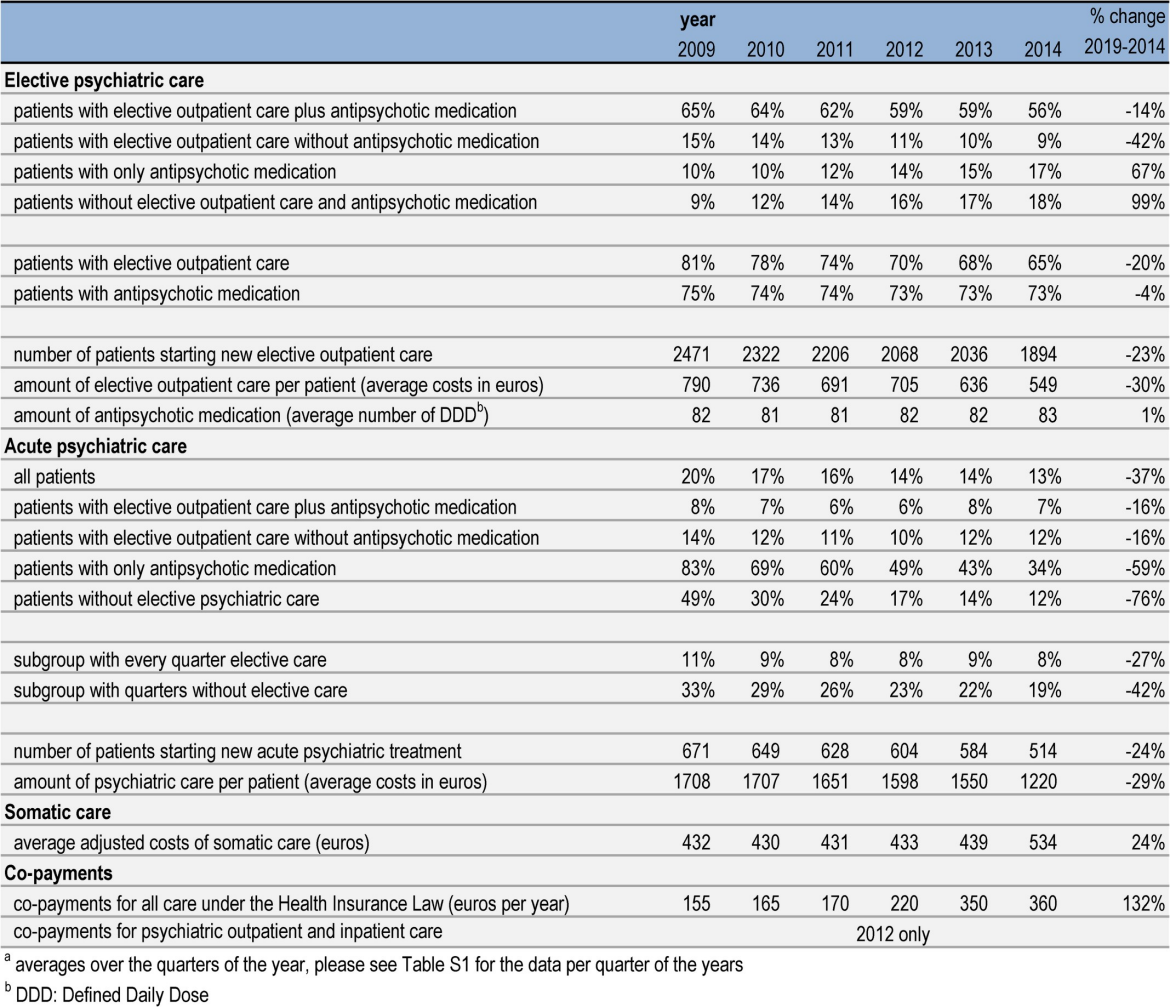
Trends of psychiatric and somatic care for patients with schizophrenia and co-payments over 2009–2014.

In the Netherlands the level of co-payment is low compared to other countries, but the level of co-payment has risen from €155 in 2009 to €360 in 2014 (Figs 2 and 3). Individual co-payments per patient are not available in our dataset. There were no co-payments concerning visits to a general practitioner.

Only during 2012 extra co-payments for psychiatric care had to be paid. These extra co-payments were €100-€200 per year for psychiatric treatment and €145 per month of inpatient care starting at the second month of treatment. Crisis treatment, involuntary treatment, and treatment of patients under 18 years of age were exempted from these co-payments[38].

### Measures

We evaluated the association between the level of co-payments and healthcare received over the years 2009–2014. Healthcare as covered by the Health Insurance Law[28,30] was divided in (i) elective outpatient psychiatric care (non-crisis psychiatric outpatient care, antipsychotic medication), (ii) acute psychiatric care (psychiatric crisis treatment and psychiatric hospitalization), and (iii) somatic care.

All measures were aggregated per quarter of the years 2009–2014. The quarters per year will be referred as q1-q4, e.g. the third quarter of 2010 as 2010q3.

Other measures are the number of patients starting new psychiatric treatments per quarter and the average amount of psychiatric care per patient, reflected by the costs of care. The costs of psychiatric care were calculated using average national prices in euros and allocated to the quarter of the starting date. The costs were adjusted for inflation using price indices for psychiatric care with 2009 as basis[46,47].

Medication was assigned to a quarter based on the pick-up day of that medication. The average amount of medication is given in Defined Daily Dose (DDD).

Somatic care, including all non-antipsychotic medication, was assigned to a quarter based on the starting day of the somatic treatment. The average amount of somatic care, reflected by the costs of care, was calculated and adjusted with cost indices for somatic care derived from all insured by Zilveren Kruis with 2009 as basis.

Continuity of elective care was defined as receiving elective psychiatric care during every quarter from 2009–2014.

### Analysis

The aim of our analysis was to test whether the abrupt and substantial rise in co-payments from 2012 on was associated with changes in the elective and acute care. Therefore, we analyzed trends in care consumption and deviations from these trends.

First, the trends in elective psychiatric care received by the patients in the cohort per quarter of the year were analyzed over the years 2009–2014, followed by the trends in acute psychiatric care, and somatic care. Next, the trends in psychiatric care for the groups with and without continuous elective care were examined. When changes in trends of care usage over time were observed these were tested by Box-Jenkins autoregressive integrated moving average (ARIMA) models[48,49]. These models describe temporal changes in trends (for an explanation please see S1 Supplement). All analyses were performed with SAS Enterprise guide 6.1 [SAS Institute Cary, NC, USA].

## Results

### Elective psychiatric care

Although the amount of received elective psychiatric care declined over follow-up, the majority of patients with schizophrenia (59%) remained in elective outpatient care over the whole period. However, the percentage of patients in a quarter receiving outpatient psychiatric care plus antipsychotic medication declined from 65% to 56% (-14%) per year over 2009–2014 (Figs 2 and 3). Outpatient psychiatric care without antipsychotic medication declined from 15% to 9% (-42%). The percentage of patients that received only antipsychotic medication increased from 10% to 17% (67% increase). Patients receiving no elective outpatient psychiatric care and no antipsychotic medication increased from 9% to 18% (99% increase). Taken together the percentage of patients with any form of elective care in a quarter declined from 91% to 82%. Outpatient psychiatric care decreased, both with regard to newly initiated treatments (-23%) and in the total amount of care (-30%). The amount of antipsychotic medication used as reflected in DDD remained almost the same over the period.

**Fig 4:**
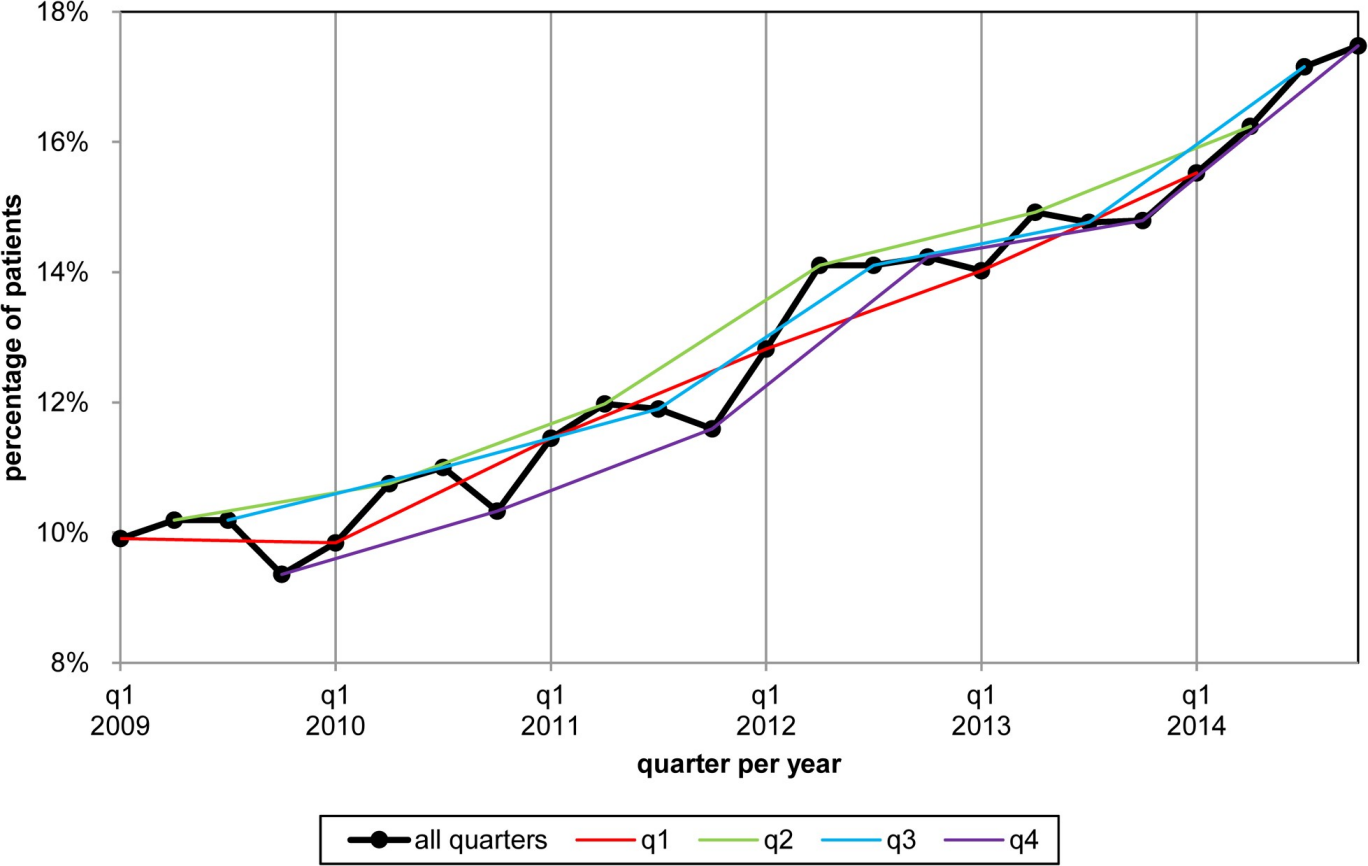
Percentage of patients with schizophrenia treated with only antipsychotic medication.

Next, we analyzed if abovementioned trends were constant or whether there were deviations from these trends. First, the increasing trend in percentage of patients treated with only antipsychotic medication shifted to a higher level in 2012q1 and in 2014q3 (S1 and S2 Tables, Fig 4).

Second, the decreasing trend in the number of patients starting new elective outpatient psychiatric care shifted to a higher level from 2012q4-2013q3. Third, the decreasing trend in amount of elective outpatient psychiatric care per patient shifted to a higher level in 2012q4.

### Acute psychiatric care

**Fig 5:**
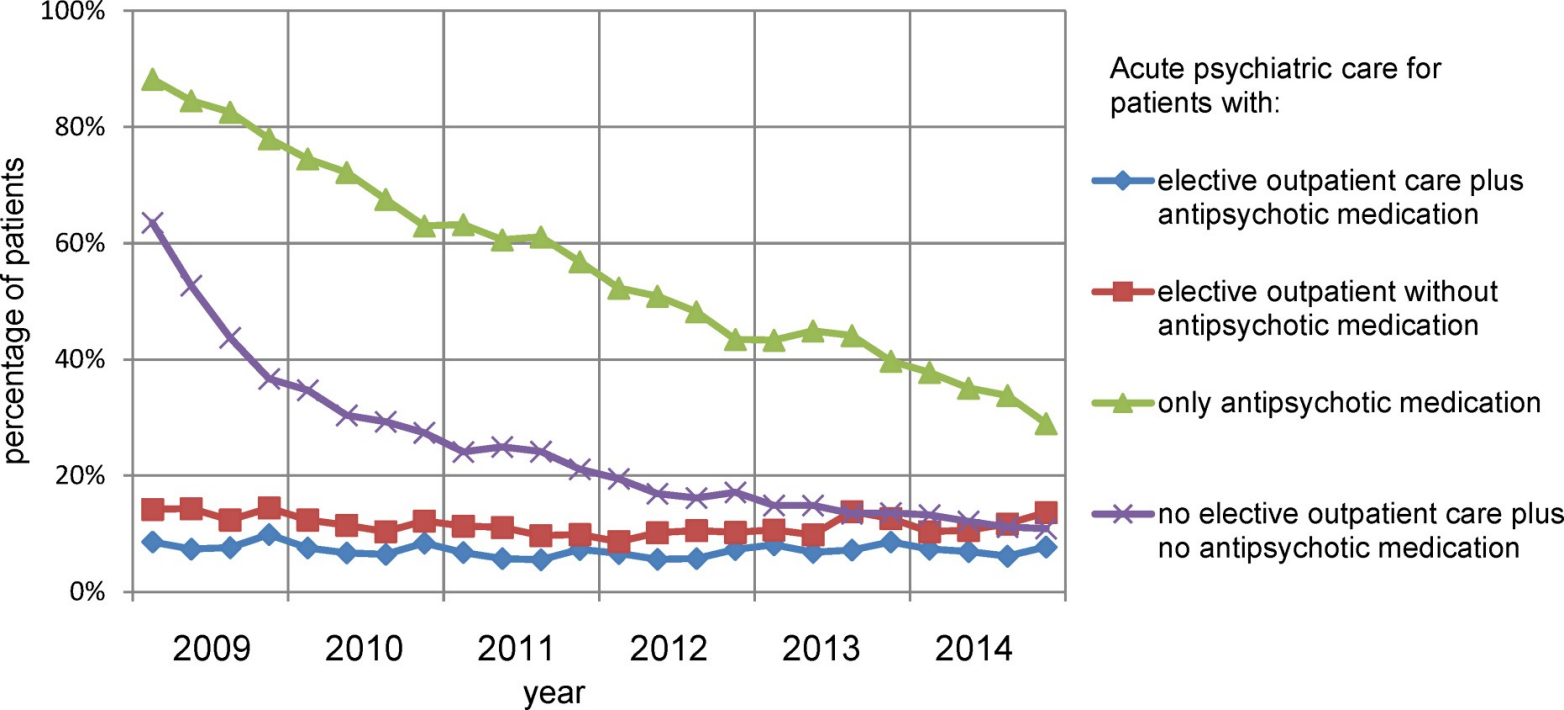
Acute psychiatric care by type of elective psychiatric care for patients with schizophrenia.

The percentage of patients needing acute psychiatric care declined from 20% to 13% (-37%) over 2009–2014 (Fig 3). The relation between elective psychiatric care and acute psychiatric care was examined. The levels of acute psychiatric care among patients with elective outpatient care, with or without antipsychotic medication, per quarter are under 15% average per year over 2009–2014 and declined with 16% from 2009–2014 (Figs 3 and 5). The level of acute psychiatric care in quarters with only antipsychotic medication was very high with an average of 83% in 2009 and declined to 34% in 2014 (-59%). In quarters without elective psychiatric care the average level of acute psychiatric care was also very high in 2009 (49%) declining to 12% (-76%) in 2014.

**Fig 6:**
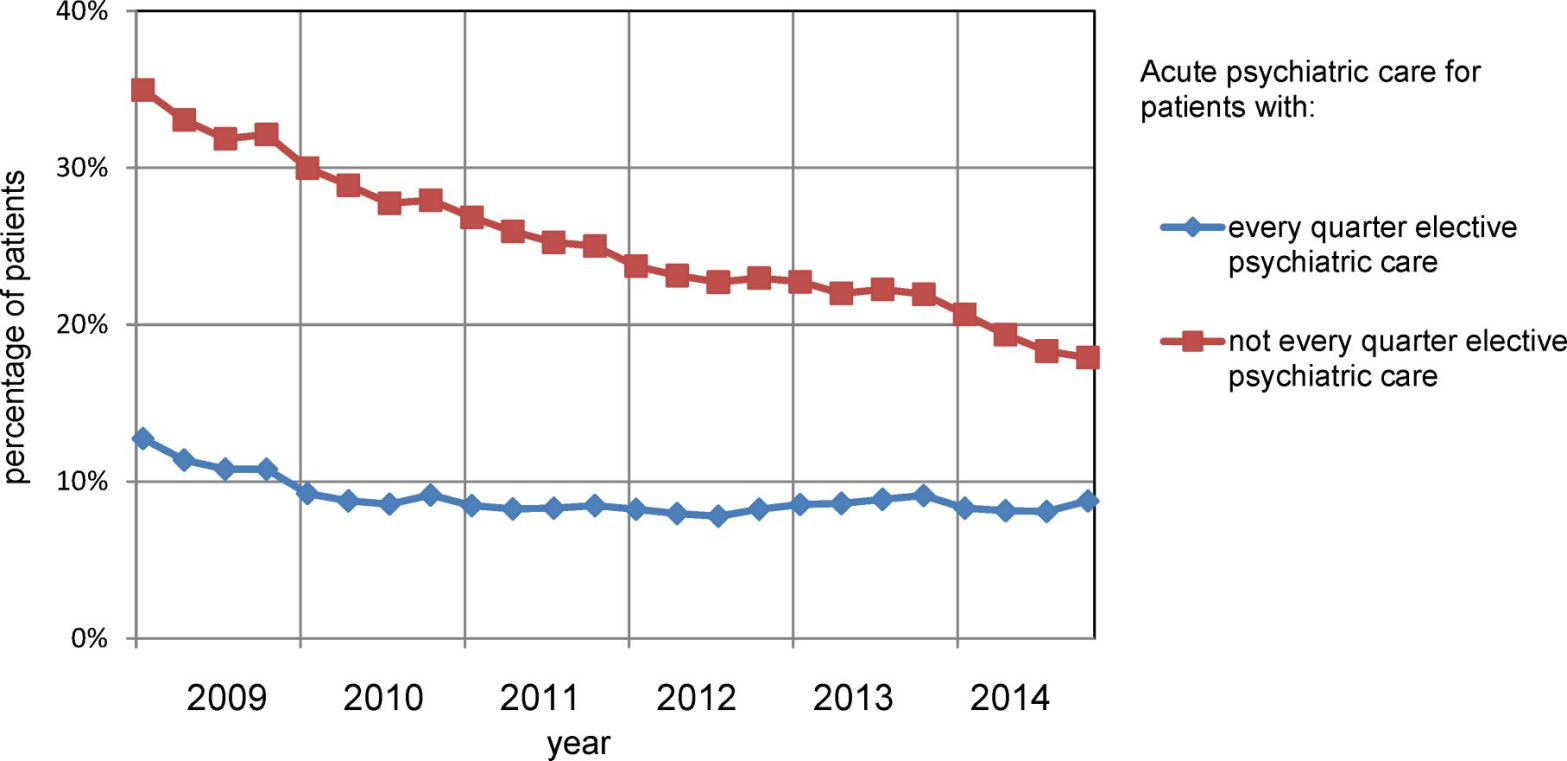
Acute psychiatric care for the subgroups with and without continuity of psychiatric care for patients with schizophrenia.

The subgroup of patients with elective psychiatric care during all quarters needed less than half the levels of acute psychiatric care as compared to the other patients over 2009–2014 (Figs 3 and 6). Acute psychiatric care decreased in both newly initiated treatments (-24%) and in the total amount of care (-29%).

Several deviations of abovementioned trends occurred. The percentage of patients with acute care showed an increased level shift from 2012q2 (S1 and S2 Tables). The patients with elective outpatient care plus antipsychotic medication shifted to a higher level of acute psychiatric care in 2012q1 and to a lower level in 2013q4. The patients with elective outpatient care without antipsychotic medication shifted to a higher level of acute psychiatric care in 2012q3. Patients with only antipsychotic medication also shifted to a higher level in 2012q3. The subgroup of patients with quarters without elective psychiatric care shifted to a higher level of acute psychiatric care in 2012q1. The number of patients starting a new episodic treatment shifted to a higher level in 2012q2 and to a lower level in 2013q4. The amount of acute psychiatric care had an increased level shift from 2012q1 and a downward level shift from 2014q1.

### Somatic care

The average amount of somatic care, including non-antipsychotic medication, per patient with schizophrenia per year rose with 24%, per quarter from €432-€534 (Fig 3) without significant deviations from this trend.

## Conclusion and discussion

Our goal was to study the healthcare received by a cohort of patients with schizophrenia during a period of substantially rising co-payments. Our most important findings are: over the whole period (2009–2014) consumption of psychiatric healthcare decreased strongly, while consumption of somatic healthcare increased. The percentage of patients receiving only antipsychotic medication increased and the percentage of patients without any elective psychiatric care also increased substantially. Use of acute psychiatric care was highest in quarters when patients received only antipsychotic medication. Continuous elective psychiatric care over the whole period was received by 59% of the patients, who showed less than half the levels of episodic psychiatric care compared to the other patients.

On top of this decreasing trend in access to psychiatric healthcare we found that the abrupt rise in co-payments from 2012 onwards coincided with relative increases in patients treated with only antipsychotic medication as well as with relative increases in acute psychiatric care.

The overall reduction in consumption of psychiatric healthcare is spectacular and much higher than expected[18,32]. The overall reduction in acute care may be explained as a result of a strongly pursued national policy to substitute episodic care with elective, planned outpatient treatment, thereby reducing institutionalization and enhancing social participation among patients[43]. However, we would have expected that a reduction in acute care would coincidence with increased elective psychiatric care. This is not what we observed. During the study period both outpatient psychiatric care consumption and episodic psychiatric care consumption decreased.

Although causal inferences from a naturalistic study should be interpreted with caution our results support the hypothesis that co-payments have unwanted effects on the appropriate healthcare use among patients with schizophrenia. There are several factors that may have mitigated the potential effects of general co-payments. For instance, health insurance companies and cities have established health insurance contracts for low-income groups with provisions to reduce the co-payments. Patients with schizophrenia have low incomes and may have profited from these arrangements. Furthermore, a number of providers did not collect the specific co-payments for psychiatric care. Had such dampening measures not been taken, the potential effects of co-payments may have been even larger.

### Strengths and limitations

We consider the following as strengths of our research. Large registry databases complement the insights provided by trials, especially about the healthcare provided and the effects on large groups at population level. Research on registry data is rare. Most of the available studies analyze one aspect of healthcare for patients with schizophrenia, for instance the use of antipsychotic medication, costs, family services, or continuity of care[5,8,10,23–25,50–54]. Our examination of the relationship between continuity of care, co-payments and episodic psychiatric care, with acute psychiatric care as a proxy of quality of care, is new and encompasses all types of care available. The strength of our data is its reliability. Registry data from health insurers in the Netherlands are comprehensive and include both mental and somatic healthcare from all healthcare providers. These data are thought to be reliable because it is important for patients, providers, and insurers that the data are correct and the National Care Authority regulates and controls the claims process thoroughly. A further strength is that, in the Netherlands, there is universal access to (compulsory) health insurance which means that only very few people are uninsured. Throughout the country there are well-developed facilities for the treatment of patients with schizophrenia. This means that there is little room for bias by access to insurance or due the availability of services.

The results of our study should be interpreted in the light of several limitations. The associations between co-payments and healthcare use may have been driven by patient characteristics (bias by indication) and the way healthcare providers have adapted to policy changes during the study. During the study period the outcome variables we studied are probably influenced by the nation-wide policy to decrease the amount of clinical care[43]. Second, because detailed information about other DSM-IV diagnoses was not available, associations between co-morbid psychiatric disorders and outcome and costs could not be analyzed. Third, the patients that remained insured by Zilveren Kruis during the whole period may be different from those who left. However, in 2008 only 240 (1.5%) were not insured that whole year by Zilveren Kruis and in the 2009–2014 period, a minority (2 458, 17%) of the patients selected in 2008 were not insured by Zilveren Kruis for the whole period. A fourth limitation is that only two outcomes in a naturalistic study are analyzed: (1) acute care, (2) amount of psychiatric and somatic care.

### Conclusion

Although the observational design of our study precludes firm causal inference, we conclude that it is highly likely that the rise in co-payments for mental health care in the Netherlands has substantially contributed to a decrease in patients accessing elective continuous outpatient care and an increase in both their using stand-alone antipsychotic medication and acute crisis or inpatient care. This is clearly and untoward effect of measures taken to control the rising costs of health care. The results are not only relevant for The Netherlands, but also interesting for other countries with intentions to raise or introduce co-payments.

We recommend that experiments are started in which co-payments are lifted or where patients are even rewarded when accessing appropriate care.

## Supporting information

S1 Supplement Methods: Analysis of deviations of trends with Box-Jenkins autoregressive integrated moving average (ARIMA) models. (PDF)

S1 Table Trends of psychiatric and somatic care and general co-payments, results of time series analysis (ARIMA) over 2009–2014. (PDF)

S2 Table Trends of psychiatric and somatic care and general co-payments, parameters of time series analysis (ARIMA). (PDF)
