# Supplementary material for: Rising co-payments coincide with unwanted effects on continuity of healthcare for patients with schizophrenia in the Netherlands
Source: PLoS One. 2019 Sep 12;14(9):e0222046. doi: 10.1371/journal.pone.0222046 (PMC6742391; doi:10.1371/journal.pone.0222046)

S1 Supplement: Methods: analysis of deviations of trends with Box-Jenkins autoregressive integrated moving average (ARIMA) models

The aim of the analysis of the trends and deviations of the trends in use of healthcare was to examine our hypothesis that the rise in co-payments coincided with a decrease in elective outpatient psychiatric care and an increase of episodic psychiatric care among patients with schizophrenia. We first inspected the trends per quarter over the follow-up period 2009-2014 graphically to observe any deviations of these trends. In our study examples of deviations of trends are that the trend continues to decrease or increase, but at a higher or lower level (upward and downward level shifts). In other words, a decreasing trend can have a change to a higher level, but still be decreasing after that point in time, see schematic examples in Figure 1.
When changes in trends of care usage over time were observed these were tested by Box-Jenkins autoregressive integrated moving average (ARIMA) models[1–5]. These models describe temporal changes in the trends. The care data showed seasonal patterns per quarter over the years, therefore the fourth difference was used to make the time series stationary. To assess the changes in trends we applied the augmented Dickey Fuller (ADF) test and the (partial) autocorrelation functions were used to assess the 'stationarity' of the models. The 'white noise' assumption was assessed by checking if the autocorrelations differ significantly from 0 (Ljung-Box statistic). Goodness of fit was tested with the Akaike Information Criterium (AIC) and Bayesian Information Criterium (BIC). By developing one ARIMA model for each of the time series there was no multiple testing and no corrections for multiple testing were necessary. All analysis was performed with SAS Enterprise guide 6.1 [SAS Institute Cary, NC, USA].

References

1. Box, G. & Jenkins, G. *Time series analysis: forecasting and control, revised edition. Holden Day* (Holden Day, 1976).

2. Helfenstein, U. Box-Jenkins modelling in medical research. *Statistical Methods in Medical Research* **5,** 3–22 (1996).

3. Box, G. & Pierce, D. A. Distribution of Residual Autocorrelations in Autoregressive-Integrated Moving Average Time Series Models. *Journal of the American Statistical Association* **65,** 1509–1526 (1970).

4. SAS Institute Inc. *SAS/ETS® 9.2 User's Guide*. *SAS Institute Inc* (SAS Institute Inc, 2008).

5. Chin, K. *et al.* Temporal Changes in Characteristics, Treatment and Outcomes of Heart Failure Patients Undergoing Percutaneous Coronary Intervention Findings from Melbourne Interventional Group Registry. *Heart, Lung and Circulation* (2018). doi:10.1016/j.hlc.2018.03.030

Figure 1: schematic examples of trends with upward and downward level shifts

Figure 1A: upward trend with upward level shift

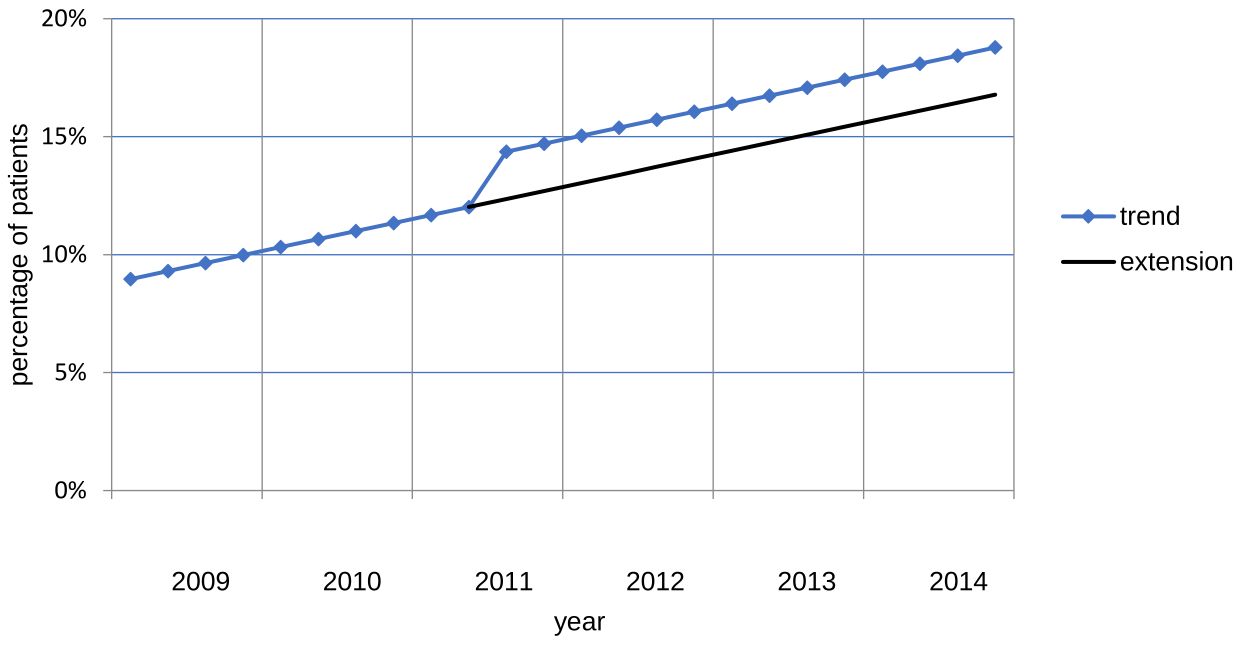

Figure 1B: downward trend with downward and upward level shift

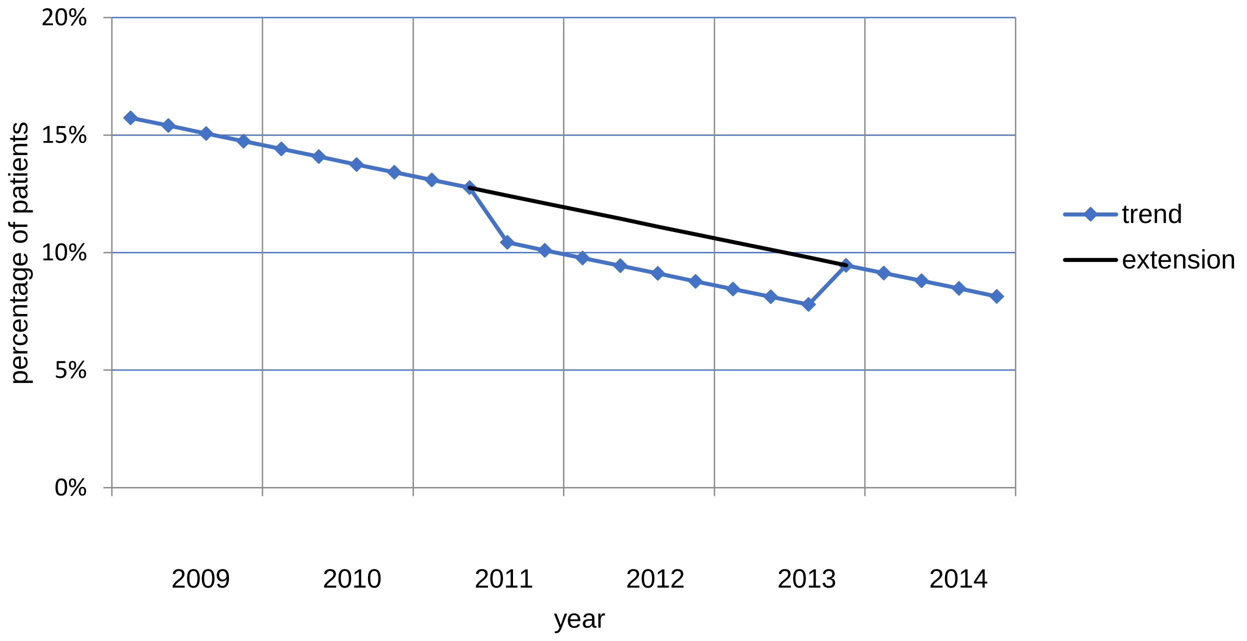

Supplement: S1 Supplement — (PDF) [file pone.0222046.s001.pdf]
