# Supplementary material for: Rising co-payments coincide with unwanted effects on continuity of healthcare for patients with schizophrenia in the Netherlands
Source: PLoS One. 2019 Sep 12;14(9):e0222046. doi: 10.1371/journal.pone.0222046 (PMC6742391; doi:10.1371/journal.pone.0222046)
Supplement: S1 Table — (PDF) [file pone.0222046.s002.pdf]

S1 Table. Trends of psychiatric and somatic care and general co-payments, results of time series analysis (ARIMA) over 2009-2014

year	2009				2010				2011				2012				2013				2014			
quarter	q1	q2	q3	q4	q1	q2	q3	q4	q1	q2	q3	q4	q1	q2	q3	q4	q1	q2	q3	q4	q1	q2	q3	q4
**Elective psychiatric care**																								
patients with elective outpatient care plus antipsychotic medication	66%	65%	64%	66%	65%	64%	63%	64%	62%	62%	61%	62%	60%	59%	59%	59%	59%	59%	58%	59%	57%	56%	55%	55%
patients with elective outpatient care without antipsychotic medication	16%	16%	16%	14%	14%	14%	14%	13%	13%	13%	13%	12%	12%	11%	11%	10%	10%	9%	10%	9%	9%	9%	9%	8%
patients with only antipsychotic medication	10%	10%	10%	9%	10%	11%	11%	10%	11%	12%	12%	12%	13%	14%	14%	14%	14%	15%	15%	15%	16%	16%	17%	17%
patients without elective outpatient care and antipsychotic medication	8%	9%	10%	10%	11%	11%	12%	13%	13%	14%	14%	14%	15%	16%	16%	16%	16%	17%	17%	17%	18%	18%	19%	19%
patients with elective outpatient care	82%	81%	80%	80%	79%	78%	77%	77%	76%	74%	74%	74%	72%	70%	70%	70%	69%	68%	68%	68%	66%	65%	64%	64%
patients with antipsychotic medication	76%	76%	75%	75%	75%	75%	74%	74%	74%	74%	73%	74%	73%	74%	73%	74%	73%	74%	73%	73%	73%	73%	72%	73%
number of patients starting new elective outpatient care	2725	1872	2035	3250	2579	1724	1929	3055	2507	1614	1854	2847	2244	1473	1721	2834	2122	1520	1885	2616	2123	1462	1702	2289
amount of elective outpatient care per patient (average costs in euros)	875	555	636	1093	810	540	615	978	780	513	571	902	742	514	590	976	657	481	568	838	628	425	470	675
amount of antipsychotic medication (average number of DDD[a])	80	83	80	84	79	82	80	82	80	82	81	82	81	82	81	84	81	83	81	84	80	83	83	86
**Episodic psychiatric care**																								
all patients	22%	20%	20%	20%	18%	17%	17%	17%	16%	16%	15%	15%	15%	14%	14%	14%	14%	14%	14%	14%	13%	13%	12%	13%
patients with elective outpatient care plus antipsychotic medication	9%	7%	8%	10%	8%	7%	6%	8%	7%	6%	6%	7%	7%	6%	6%	7%	8%	7%	7%	9%	7%	7%	6%	8%
patients with elective outpatient care without antipsychotic medication	14%	14%	12%	14%	12%	11%	10%	12%	11%	11%	10%	10%	9%	10%	11%	10%	11%	10%	14%	13%	10%	11%	12%	14%
patients with only antipsychotic medication	88%	85%	83%	78%	74%	72%	68%	63%	63%	61%	61%	57%	52%	51%	48%	43%	43%	45%	44%	40%	38%	35%	34%	29%
patients without elective psychiatric care	64%	53%	44%	37%	35%	30%	29%	27%	24%	25%	24%	21%	19%	17%	16%	17%	15%	15%	13%	14%	13%	12%	11%	11%
subgroup with every quarter elective care	13%	11%	11%	11%	9%	9%	9%	9%	8%	8%	8%	8%	8%	8%	8%	8%	9%	9%	9%	9%	8%	8%	8%	9%
subgroup with quarters without elective care	35%	33%	32%	32%	30%	29%	28%	28%	27%	26%	25%	25%	24%	23%	23%	23%	23%	22%	22%	22%	21%	19%	18%	18%
number of patients starting new episodic psychiatric treatment	725	590	610	760	637	504	516	678	575	461	439	574	518	450	507	629	644	499	564	646	543	485	464	562
amount of psychiatric care per patient (average costs in euros)	1742	1525	1577	1986	1737	1304	1365	1794	1468	1075	1262	1661	1596	1091	1359	1827	1505	1121	1328	1764	1336	940	1133	1472
**Somatic care**																								
average adjusted costs of somatic care (euros)	410	444	435	439	400	448	445	462	424	460	467	464	485	449	450	468	507	526	524	485	520	525	550	539
**Co-payments**																								
co-payments for all care under the Health Insurance Law (per year in euros)	155				165				170				220				350				360			
co-payments for psychiatric outpatient and inpatient care													*psychiatric co-payments*											
**Price and cost indices for psychiatric and somatic care**																								
price index psychiatric care (treatment)	100				98				99				100				104				115			
price index psychiatric care (hospital bed)	100				98				99				83				85				86			
cost index somatic care	100	96	94	98	100	97	96	101	105	100	98	102	102	105	102	108	109	108	106	111	110	110	109	113

year	2009				2010				2011				2012				2013				2014			
quarter	q1	q2	q3	q4	q1	q2	q3	q4	q1	q2	q3	q4	q1	q2	q3	q4	q1	q2	q3	q4	q1	q2	q3	q4
**Deviations in trends: p-values, Level Shifts and Additive Outliers[b]**																								
**Elective psychiatric care**																								
patients with elective outpatient care plus antipsychotic medication																								
patients with elective outpatient care without antipsychotic medication																								
patients with only antipsychotic medication													0,0362			0,0071							0,0284	
patients without elective outpatient care and antipsychotic medication																								
patients with elective outpatient care																								
patients with antipsychotic medication																								
number of patients starting new psychiatric treatment																0,003			0,0459			0,0328		
amount of psychiatric care per patient (average costs in euros)																c								
amount of antipsychotic medication (average number of DDD)																								
**Episodic psychiatric care**																								
all patients														<0,0001										
patients with elective outpatient care plus antipsychotic medication													<0,0001							0,0004				
patients with elective outpatient care without antipsychotic medication															<0,0001									
patients with only antipsychotic medication															0,002									
patients without elective psychiatric care																								
subgroup with every quarter elective care																								
subgroup with quarters without elective care													<0,0001											
number of patients starting new psychiatric treatment														<0,0001						0,0021				
amount of psychiatric care per patient (average costs in euros)													<0,0001								0,0003			
**Somatic care**																								
average adjusted costs of somatic care (euros)																								

[a] DDD: Defined Daily Dose

[b] results of testing deviations of trends with Arima are indicated in the table with: upwards level shift; downwards level shift; additve outlier

[c] 2012q4: additive outlier (p=0,0003) and upwards level shift (p-0,0435)
