# Supplementary material for: Rising co-payments coincide with unwanted effects on continuity of healthcare for patients with schizophrenia in the Netherlands
Source: PLoS One. 2019 Sep 12;14(9):e0222046. doi: 10.1371/journal.pone.0222046 (PMC6742391; doi:10.1371/journal.pone.0222046)
Supplement: S2 Table — (PDF) [file pone.0222046.s003.pdf]

S2 Table. Trends of psychiatric and somatic care and general co-payments, parameters of time series analysis (ARIMA)

	parameters time-series analysis (ARIMA)[a]			
	AIC	mu	ma1	
**Elective psychiatric care**				
patients with elective outpatient care plus antipsychotic medication	-147	<0,0001	0,0176	
patients with elective outpatient care without antipsychotic medication				no deviations
patients with only antipsychotic medication	-156	<0,0001	0,0017	
patients without elective outpatient care and antipsychotic medication				no deviations
patients with elective outpatient care				no deviations
patients with antipsychotic medication				no deviations
number of patients starting new psychiatric treatment	236	<0,0001	0,0054	
amount of psychiatric care per patient (average costs in euros)	207	0,0189	0,0024	
amount of antipsychotic medication (average number of DDD[b])				no deviations
**Acute psychiatric care**				
all patients	-137	<0,0001	0,0002	
patients with elective outpatient care plus antipsychotic medication	-154	<0,0001	0,0198	
patients with elective outpatient care without antipsychotic medication	-124	<0,0001	0,0002	
patients with only antipsychotic medication	-93	<0,0001	0,0009	
patients without elective psychiatric care				no deviations
subgroup with every quarter elective care				no solution
subgroup with quarters without elective care	-131	<0,0001	0,0012	
number of patients starting new acute psychiatric treatment	202	<0,0001	0,0099	
amount of psychiatric care per patient (average costs in euros)	234	<0,0001	0,0243	
**Somatic care**				
average adjusted costs of somatic care (euros)				no deviations

[a] AIC: Akaike information criterium, mu: mean estimate, ma1: moving average estimate

[b] DDD: Defined Daily Dose
